# Classification of decomposed neural data in memory networks and LLM-based stimuli processing

**DOI:** 10.1007/s11682-026-01148-4

**Published:** 2026-04-18

**Authors:** Muhammad Shahzaib, Salma Zainab Farooq, Eric H. Schumacher, Shella D. Keilholz, Sadia Shakil

**Affiliations:** 1 Department of Electrical Engineering, Institute of Space Technology, Islamabad, Pakistan; 2 School of Psychology, Georgia Institute of Technology, Atlanta, GA USA; 3 Wallace H. Coulter Department of Biomedical Engineering, Emory University/ Georgia Institute of Technology , Atlanta, GA USA; 4 Department of Biomedical Engineering, The Chinese University of Hong Kong, Hong Kong SAR, China

**Keywords:** Naturalistic stimuli, Functional connectivity (FC), Memory recall, Low-rank approximation, Automatic scene segmentation, Large Language Model (LLM)

## Abstract

Naturalistic paradigms, where participants are exposed to real-world stimuli (e.g., narratives) are important in memory research. They may provide a more complete assay of human memory processes and their underlying mechanisms compared to controlled task-based paradigm. In these paradigms, stimuli are continuous and do not have well-defined scene boundaries. Defining scene boundaries based on narrative events such as plot twists or character developments is crucial as these moments are known to influence memory recall. Aligning neural activity with such boundaries helps in studying the dynamics of memory recall during narrative experiences. However, segmenting narratives based on memory-driven cues is difficult due to the continuous flow of events. To overcome this, we developed an automatic scene segmentation technique using large language models (LLMs). The LLMs segment narratives into meaningful scenes offering a consistent unbiased method for recall-based segmentation. These segments are then used to analyze brain dynamics from fMRI data. In our study, 180 participants listened to four different stories while undergoing fMRI. Functional connectivity (FC) was computed based on the LLM-derived scene segments. For memory recall analysis, classification models were trained using participants’ recall scores as labels. To further understand the neural basis of memory, FC matrices were decomposed into shared (low-rank) and individual (idiosyncratic) components. Classification results demonstrate that LLM-based segmentation effectively defines scene boundaries and that memory recall is not solely tied to common or idiosyncratic activity. This approach offers a robust framework for exploring brain-behavior relationships in naturalistic memory research.

**Supplementary Information:**

The online version contains supplementary material available at 10.1007/s11682-026-01148-4.

## Introduction

Accurately segmenting continuous naturalistic experiences into meaningful cognitive units, such as scenes or events, is a fundamental prerequisite for studying perception, memory, and neural dynamics in both cognitive neuroscience and psychology (Zacks & Sargent, 2010). Event segmentation theory posits that individuals discretize ongoing experiences at boundaries where perceptual, conceptual, or narrative shifts occur, shaping how information is encoded and subsequently recalled (Baldassano et al., 2017). Traditionally, the identification of such boundaries has relied on manual annotations from human raters (Chen et al., 2017; Zadbood et al., 2017), a process that is not only labor-intensive and costly but also introduces subjectivity and inter-rater variability. Segmenting narratives into scenes is valuable for both understanding stories and for computational processing. It creates smaller, coherent units that can be analyzed individually and compared across a corpus (Guhr et al., 2025). In general, information learned just before a scene change is more likely to be forgotten than information within the same scene (Michelmann et al., 2023). Empirical studies confirm that people’s recall of a story is structured by its event segmentation – free recall tends to be clustered by scenes (items from the same event are recalled together) (Karagoz et al., 2025). Interestingly, while event boundaries help organize memory, they can also impair memory for the immediately preceding content: one study found that memory for words presented right after a boundary was worse than for words in the middle of a scene (Karagoz et al., 2025). On the other hand, event boundaries seem to serve as access points for memory search; when trying to remember a long sequence, people “skip” through events at the boundaries to locate the target memory (Michelmann et al., 2023). Overall, these traditional perspectives underscore that scenes are cognitively meaningful units – narrative comprehension and recall are deeply influenced by how a story is segmented into events. To address these challenges, recent work has begun to explore the use of large language models (LLMs) as scalable, automated tools for event segmentation in narrative texts. In a recent work, Michelmann et al. (2025) demonstrated that LLMs, specifically GPT-3, can segment narrative texts into meaningful events with a level of agreement comparable to human annotators. Using a simple zero-shot prompt, GPT-3-generated event boundaries showed significant alignment with human consensus annotations. Subsequent studies have validated and expanded this approach using newer LLMs (Panela et al., 2025) demonstrating that both proprietary and open-source LLMs could automate event segmentation in a way that reflects normative human boundaries. The application of LLMs for event segmentation thus addresses long-standing limitations of manual annotation by providing a scalable, reproducible, and less biased alternative.

However, the approach of using a single model for such segmentation has a major limitation that opens the door for improvement. The use of a single model such as (GPT-3) or other newer LLMs, while providing effective automated event segmentation, inherently reflects the linguistic and cognitive biases of the model’s architecture and training data. This approach poses questions on generalizability of LLM-derived boundaries across languages, genres, and modalities (e.g., audio vs. text), as well as the influence of model architecture and training data on segmentation strategies (Fenerci et al., 2025). Developing an algorithm using more than one LLM for extracting segment boundaries would solve this generalizability issue.

Furthermore, despite their promise, applications of LLMs for event segmentation, using them to segment events based on a cognitive process such as memory recall and associating them with neural dynamics remain underexplored. Recall scores, obtained through the behavioral tests administered after an experiment, offer a direct measure of memory formation during naturalistic stimuli. These scores derived from participants’ verbal or written retelling of the events reflect how well the events were encoded and segmented in the brain. Accurately aligned event boundaries are crucial to anchor these recall assessments to specific stimulus segments, enabling a meaningful mapping between neural responses and memory performance. In naturalistic paradigms, where stimuli unfold continuously over time, misaligned or imprecise segmentation of events can lead to mixing of neural signals from different cognitive contexts, thereby diluting relevant connectivity patterns. This is particularly critical in classification (Pan et al., 2022), where scene-level labels guide both the extraction of functional connectivity (FC) matrices and the training of predictive models, making the accuracy of temporal alignment essential for reliable brain–behavior mapping. Building upon the necessity for precise temporal alignment in FC analyses, authors in Pan et al. (2022) highlight that capturing dynamic changes in brain connectivity over time is crucial for accurate classification. This underscores the importance of aligning FC analyses with the temporal structure of stimuli to effectively capture meaningful brain-behavior relationships.

One of the most common methods to acquire and analyze brain activity under naturalistic stimulation is functional MRI (fMRI). fMRI is a non-invasive neuroimaging technique that measures brain activity by detecting changes in the blood flow to the brain during some stimulation or at rest. The authors in Zadbood et al. (2017) offers a framework to understand how different parts of the brain communicate and coordinate during complex cognitive activities using fMRI. The study of brain function using FC during naturalistic stimuli such as film clips and spoken narratives offers a more ecologically valid approach to studying memory formation (Chen et al., 2017; Zadbood et al., 2017). These paradigms capture the complexity of real-world experiences, where the formation of new memories often intertwines with personal and preexisting ones (Rugg & Vilberg, 2013).

Studies also reported that the neural activity during these stimuli is dependent on the contents of the stimuli evoking a common response in the subjects as well as each subject’s own idiosyncratic response (Finn et al., 2020) based on their personality, background, and other characteristics (Miller et al., 2009). These findings make it important to study neural dynamics of both common (stimuli-induced) and idiosyncratic (individual) brain activity of the subjects in memory-related studies to explore which of these components associate more with memory recall. By decomposing FC matrices in these two parts, we can have a more nuanced understanding of how shared and individual neural responses contribute to memory recall. A recent study (Ting et al., 2021) has proposed an algorithm to breakdown the FC matrices into these two components and by separating these components, this algorithm provides a comprehensive view of both group-level dynamics and individual differences in neural activity.

In this study, we propose an algorithm using five different state-of-the-art LLMs to segment narrations into scenes. Our algorithm is more generalizable compared to other single-LLM-based algorithms and expected to perform better than them for these scenes segmentation. Using the segmented scenes and subjects’ recall scores, we generate memory-recall labels for the scenes. We then use these labels to classify actual FC, common FC, and idiosyncratic FC using simple machine learning techniques. The main contributions of this study are as follows:

1. Development of a LLM-based narrative scene segmentation algorithm using text and recall questions of narrations.
2. Classification of scene-wise memory recall from brain functional connectivity by using the generated segmentation and subjects’ recall scores.
3. Comparison of full, common, and idiosyncratic classifications.

## Methodology

**Fig. 1:**
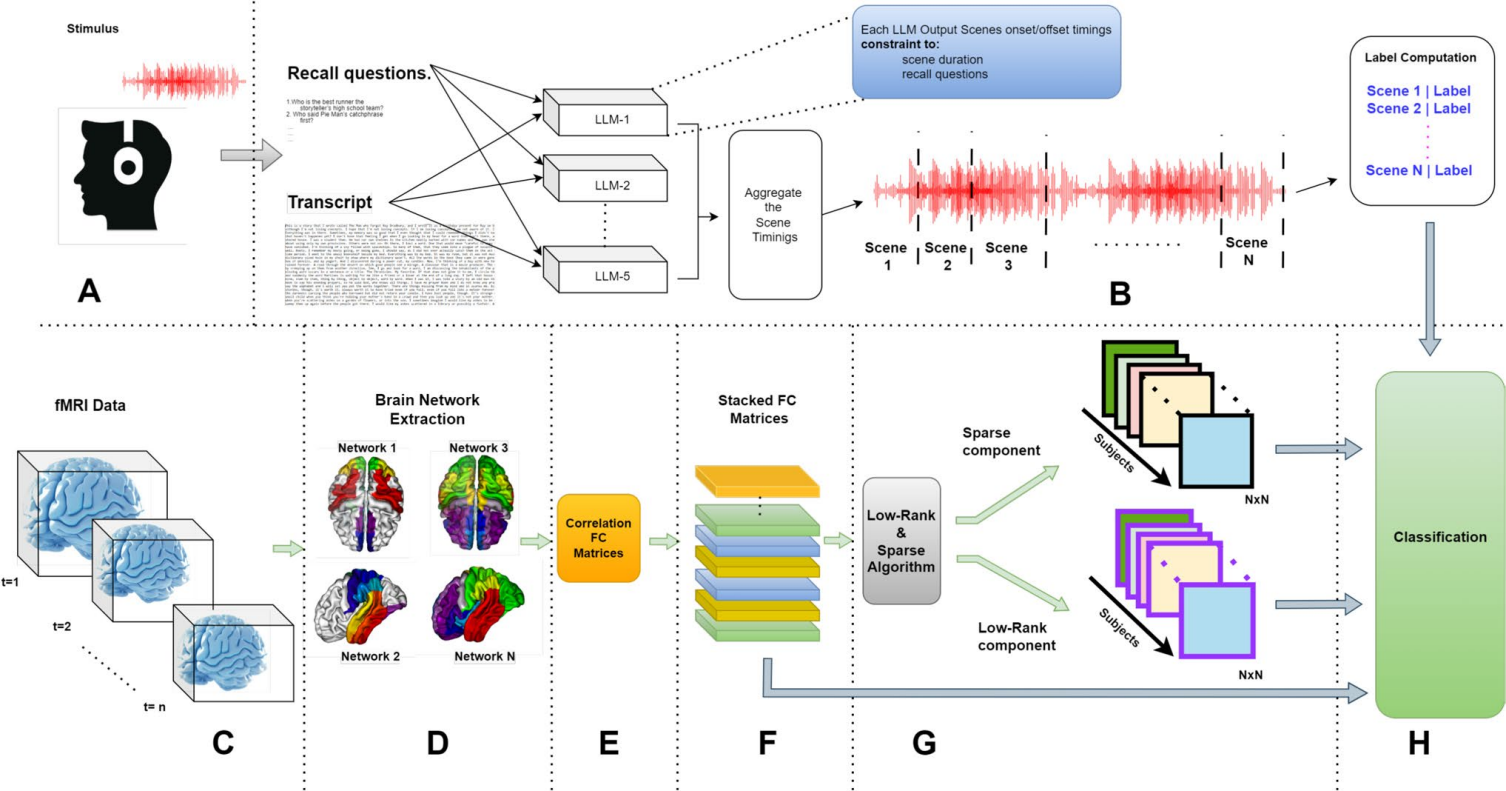
Block Diagram. Classification of recall (behavioral measure) scores through FC (neural data) matrices. These FC matrices are computed based on the scenes’ timings (narrative data) computed by proposed LLM-based algorithm. **A.** Narrative stimuli. **B.** Apply LLMs for stimuli scene segmentation, transcript and recall questions are provided to the LLMs.Corresponding recall labels are calculated based on LLMs parcellated scenes. **C.** 4D fMRI obtained from narrative datasets. **D.** Extract multiple networks. **E** & **F.** Compute FC matrices of all subjects and stack them together by concatenation. **G.** Apply Low-rank algorithm to compute decomposed sparse and low-rank components from FC matrices (each matrix has same N2 dimension as original FC matrix. **H.** Apply classification on three different FC matrices i,e Raw-FC, Low-rank component and idiosyncratic (sparse) component

In this study, we used LLMs to parcellate narrative stimuli in association with memory recall and use this segmentation to classify memory recall from the activity of memory-recall-related brain regions using functional connectivity. The block diagram of the study flow is shown in Fig. 1.

### Data

#### Stimuli

In this study four audio narrative stimuli are used, ’Pie Man (PNI)’, ’Running from the Bronx’, ’I Knew You Were Black’, and ’The Man Who Forgot Ray Bradbury’ (Nastase et al., 2019). The “Pie Man (PNI)” audio clip had a total length of 415 seconds (about 7 minutes). It began with a brief silent segment, with the narrative itself starting at 0:09 and finishing at 6:49, giving an effective story duration of 400 seconds (267 TRs). An additional 7 seconds of silence followed the end of the story. The “Running from the Bronx” stimulus lasted 561 seconds (approximately 9.4 minutes). Similar to the previous clip, it opened with a silent interval; the story began at 0:15 and concluded at 9:11, resulting in 536 seconds of actual narrative content (358 TRs). After the story, there was a 10-second silent period. The fMRI runs associated with the “Pie Man (PNI)” and “Running from the Bronx” stories consisted of 294 TRs (441 seconds) and 390 TRs (585 seconds), respectively. In both cases, the stimulus presentation started after the first 8 TRs (12 seconds) of scanning. The combined length of all stimuli was 43 minutes. There were total 184 subjects, four participants were excluded, three due to missing data caused by technical equipment failure, and one due to the absence of recall data, resulting in a final sample of 180 subjects, who listened to one of the four narrations (45 per narration, mean age 23$\pm {7.55}$ years, 52 males). After the experiment, the subjects completed individual questionnaires, consisting of multiple-choice and fill-in-the-blank questions for each story to assess comprehension, memory, and understanding after scanning. There were 25 recall questions for each of two narrations (’I Knew You Were Black’ and ’The Man Who Forgot Ray Bradbury’) and 30 each for the other two (’Pie Man (PNI)’ and ’Running from the Bronx’).

#### fMRI Data

The fMRI data was collected at the Princeton Neuroscience Institute Scully Center for Neuroimaging. Upon reaching the fMRI center, participants were asked to fill out a brief demographic form along with a standard MRI safety questionnaire. They were instructed to listen carefully to the story, stay as still as possible, and keep their eyes open during the fMRI scan, all the subjects were scanned in fMRI scanner during each story. The auditory story stimuli were delivered via MRI-compatible insert earphones, headphones or foam padding were placed over the earphones to reduce scanner noise. A 3T Siemens Magnetom Prisma having 64-channel head coil was used for fMRI data acquisition. Functional images were acquired in an interleaved fashion. TR = 1500 ms, TE = 31 ms, resolution = 2.5 $\times$ 2.5 mm, slice thickness 2.5 mm, matrix size = 96 $\times$ 96, FoV = 240 $\times$ 240 mm, flip angle = 67$^{\circ }$, bandwidth = 2480 Hz/Px, in-plane, 48 axial slices. T1-weighted structural images were also acquired using a high-resolution single-shot MPRAGE sequence. TR/TE/TI = 2530/3.3/1100 ms, flip angle = 7$^{\circ }$, in-plane,FoV = 176 $\times$ 256 $\times$ 256 mm, 176 sagittal slices resolution 1.0 $\times$ 1.0 mm, slice thickness 1.0 mm, matrix size = 256 $\times$ 256 (Nastase et al., 2019).

### Stimuli processing

#### Recall based stimuli segmentation

We utilize five state-of-the-art LLMs (ChatGPT 4.1 (OpenAI, 2025), Anthropic Claude 3 (Anthropic, 2024), Perplexity (OpenAI, 2024), Microsoft MS co-pilot (Microsoft, 2025), and Meta’s Llama 3.1 (Meta, 2025) in this study for recall-based stimuli segmentation representing a significant innovation in objective and unbiased recall-based scene segmentation. All of these LLMs are trained on vast amounts of text data and can recognize subtle narrative patterns, such as foreshadowing or tonal shifts, without the subjective judgment that may arise in human annotators. Thus by leveraging the strengths of multiple LLMs, we could mitigate the limitations inherent in human-annotated segmentation, which are often prone to subjective bias due to personal perspectives, cultural backgrounds, and emotional responses.

**Algorithm 1:**
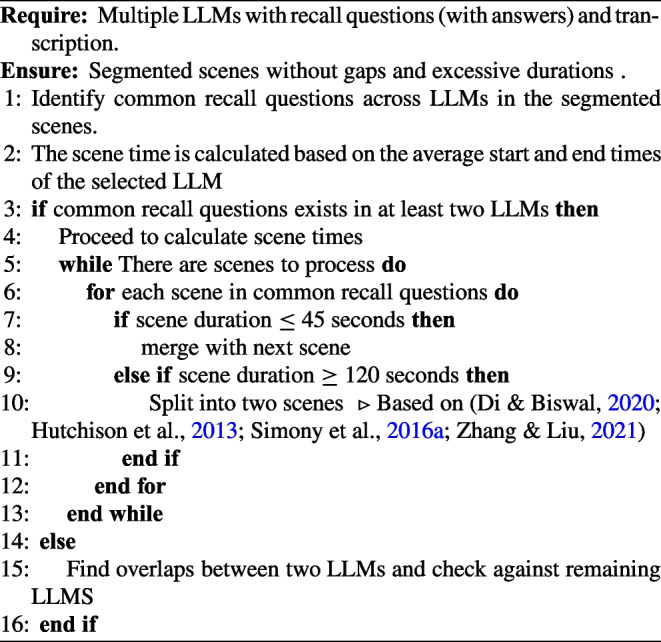
Stimuli segmentation algorithm.

The decision to use multiple LLMs for scene segmentation was deliberate, ensuring that any biases or limitations specific to individual models would be averaged out. The pseudo code outlined in Algorithm 1 explains the steps of identifying the common narrative boundaries across these LLMs. The algorithm involves the following steps:

- **Transcription and Recall Question Retrieval:** Each LLM retrieves the transcription of the story, which provides contextual information about the narrative. In addition to the transcript, each LLM also retrieves its corresponding recall questions, which are specific queries related to key events or scenes in the text. These recall questions serve as an indicator of scene boundaries (later these recall questions are used to compute recall scores for each segment).
- **Scene Identification and Segmentation:** Each LLM identifies scenes from the transcription and associated recall questions. The duration of each scene is checked to ensure it falls within a predefined range (45 seconds to 120 seconds) based on previous studies (Chen et al., 2017; Di & Biswal, 2020; Hutchison et al., 2013; Simony et al., 2016a; Zhang & Liu, 2021). The prompt specifies that the scenes should be continuous, with no time gaps between them.
- **Scene Selection:** Select common scenes from at least two LLMs based on shared recall questions.
- **Final Scene Duration Calculation:** Calculate the duration of the final scene by averaging the onset and offset timings of the selected scenes of all LLMs from the previous step.

The benefits of this approach are multifaceted:

- **Objectivity**: By leveraging LLMs, we can minimize the impact of subjective biases and ensure that the scene segmentation is based on an objective analysis of the narrative structure in a fair and consistent manner.
- **Wider Generalization:** Diverse training data in each LLM enhances both generalization and the identification of scenes.
- **Efficiency**: This approach streamlines the process of scene segmentation by human annotators, enabling researchers to quickly and accurately segment stimuli without the need for extensive human annotation.

The resulting segments from the recall-based LLM-driven stimuli segmentation process are expected to be associated with brain activity of memory related regions. In line with prior work (Di & Biswal, 2020; Hutchison et al., 2013; Simony et al., 2016a; Zhang & Liu, 2021), we constrained scene durations within 45–120 seconds to balance temporal resolution against the need for stable connectivity estimates, ensuring scenes were neither too brief for reliable FC estimation nor too long to miss meaningful context shifts

Objectively segmentation of narratives into distinct scenes (each corresponding to stimulus boundaries where related information is encoded) provides a fine grained framework for understanding how memory-related cognitive processes are linked to specific narrative events. Each subject has multiple recall scores (one recall score against each question) and each scene identified by the LLM-based segmentation contains more than one question, so to produce the scene-level recall measure we have averaged the recall scores within each scene, which serves as a behavioral label for classification. This direct link between brain activity patterns and specific cognitive or behavioral units (scenes or events) highlights the importance of understanding how the brain processes and recalls information in naturalistic settings.

### fMRI Processing

#### Preprocessing

We use AFNI software to preprocess fMRI data and use the standard pre-processing pipeline comprising slice time correction, motion correction, co-registration, normalization (MNI152), and smoothing.

#### Brain regions extraction

We use an extended version of Glasser Atlas (HCPex) (Huang et al., 2022). The Default Mode (DMN) and Auditory network (Seitzman et al., 2020) regions were extracted since these two networks are reported to be involved in speech perception (Brandman et al., 2021; Simony et al., 2016a, b; Song et al., 2023; Ting et al., 2021). Details of these two networks can be found in Supplementary Table 2.

#### Functional connectivity

We compute FC using Pearson’s correlation coefficient for each LLM-driven narration segmentation. We compute pairwise FC for all ROIs of both DMN and Auditory network for further analysis.

#### FC decomposition

The FC matrices provide an overall picture of brain functionality and cannot separate the stimuli-induced and idiosyncratic functional patterns of each subject. For this reason, the low-rank plus sparse (L+S) decomposition is used (Ting et al., 2021). The low-rank component exploits shared neuronal activity among subjects in terms of network connectivity directly rather than the low-level Blood Oxygenation Level-Dependent (BOLD) responses in ISFC (Ting et al., 2021). The algorithm proposed in Ting et al. (2021) decomposes each subject’s FC matrix in subject-specific (sparse) idiosyncratic component and stimulus-induced (low-rank) common component such that Eq. 1 Ting et al. (2021):

1 $$\begin{aligned} Z_t = L_t + S_t, \quad {\forall }_t = 1, \ldots , T \end{aligned}$$

where $Z_t$ is the input concatenated FC matrix of *M* subjects of size $R^2$, where *R* is the number of ROIs and *t* is the scene number such that $t \in \{1, \dots , T\}$ for a total of *T* scenes . This input FC is decomposed into low-rank $L_t$ plus sparse $S_t$ matrices. We perform this for all *T* scenes for dynamic analysis of the brain functionality.

### Classification

After constructing the FC matrices from the fMRI data, we performed classification tasks to evaluate the ability of different machine learning models to differentiate between the target classes (memory recall scores computed using LLM-scene segmentation). We employed three machine learning algorithms: Logistic Regression (LR), Random Forest (RF), and Support Vector Machine (SVM). We performed classification on the following FC matrices described in Table 1. For each method, all the FC matrices were stacked (concatenated) together before classification.

**Table 1:** Classification types

Method	Input to classifier
FC based classification	Stacked FC matrices of all subjects.
Low-rank classification	Stacked FC matrices, decomposed into low-Rank matrices
Idiosyncratic classification	Stacked FC matrices, decomposed into idiosyncratic matrices

First, we performed classification on FC matrices and analyze the results. Then classification was done on the decomposed FC matrices, the low-rank and Idiosyncratic components. The classification on decomposed FC allowed us to investigate whether decomposition could enhance classification performance by isolating distinct signal patterns in the data. The classification process is carried out as follows: The FC matrices were reshaped into feature vectors to serve as input to the classifiers. The input matrix has a shape of $(W, M, R^2)$ having *W* stimulus scenes/windows for *M* subjects, each matrix (component) has a size of $R^2$ with *R* number of ROIs. Each matrix, originally in the form of a symmetric 2D array, was converted into a one-dimensional feature vector by extracting its lower triangular values (excluding the diagonal). We extracted $r= [(R^2-R)/2]$ unique values from the lower triangle of each matrix. This transformation ensured that only unique connections were used as features. We did not apply explicit feature selection, instead all unique FC edges were used as features for the classifiers. $l_2$ regularization was used for both logistic regression and SVM classifiers.The classification was also performed using label shuffling in order to validate the scene alignments with correct recall labels.

## Results

### Recall based stimuli segmentation

Dynamic FC analyses often employ sliding time windows to capture dynamic interactions between brain regions. The effectiveness of these analyses hinges on aligning the windows precisely with the onset and offset of cognitive events or stimulus scenes.

**Fig. 2:**
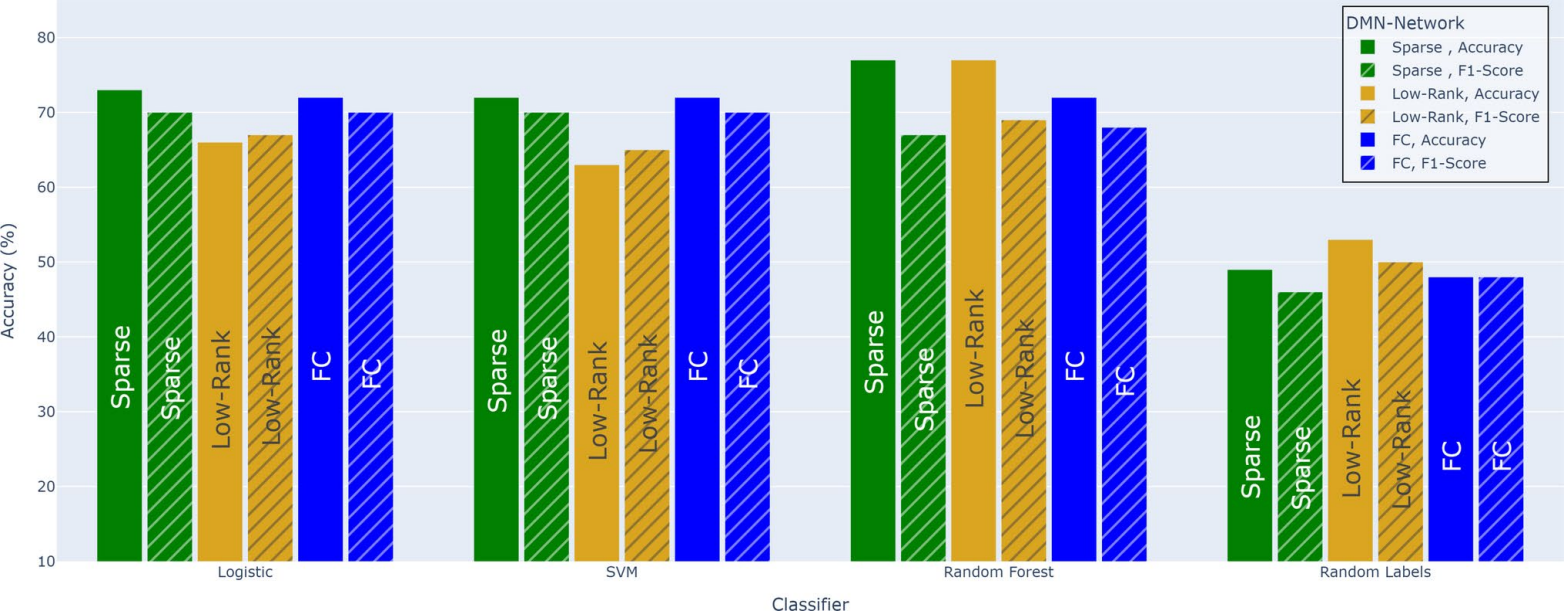
Classification comparison of decomposed FC matrices for DMN network

**Fig. 3:**
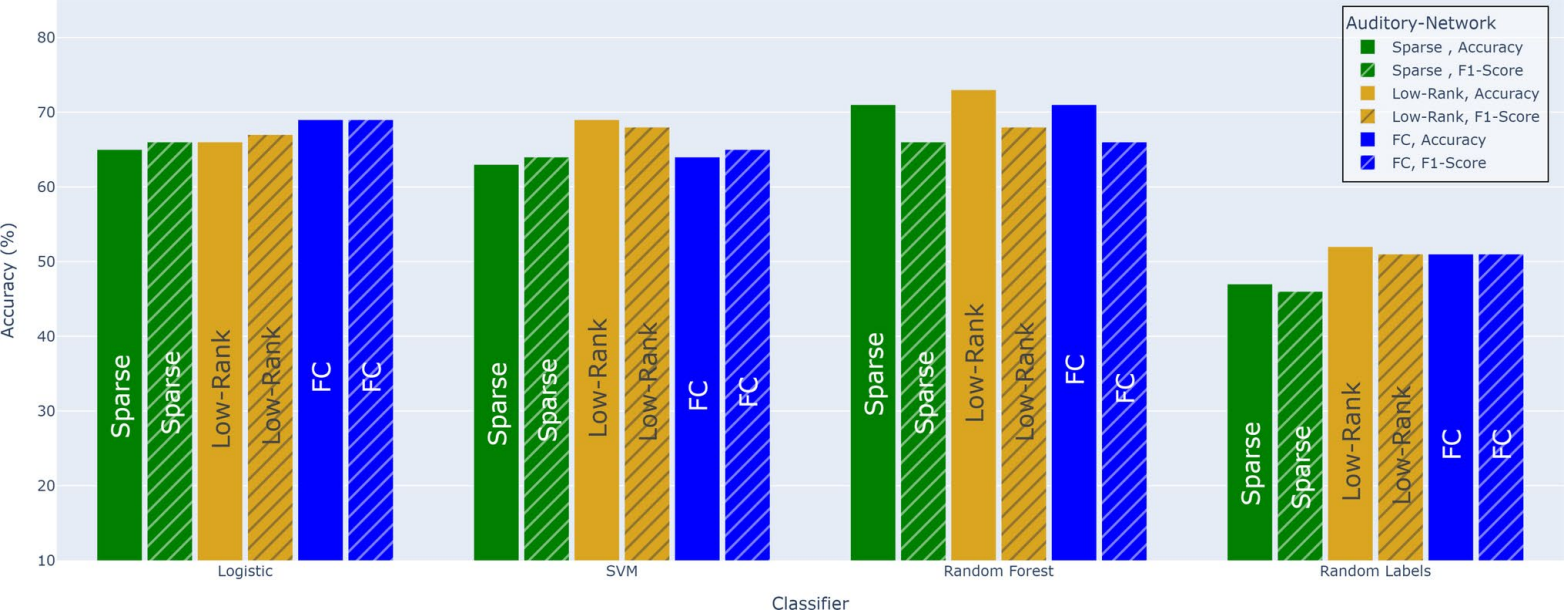
Classification comparison of decomposed FC matrices for Auditory network

**Fig. 4:**
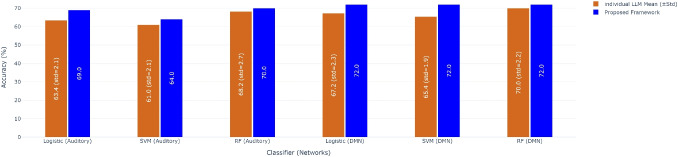
Classification accuracy comparison: individual LLMs vs proposed framework for Auditory and DMN network

We incorporated five LLMs while providing the recall questions to them along with the transcription, based on which the scene’s onset and offset timings are calculated, as described in pseudo Algorithm 1. The context window for each LLM is large enough to accommodate whole data at once. The LLMs process the text in a form of tokens, a single token is approximately equal to 0.75 words.^1^ Out of the four narrations, the “The Man Who Forgot Ray Bradbury” had the highest length with approximately 3,500 tokens, while the “Pie Man (PNI)” narration had the lowest length with around 2,050 tokens. A total of 32 scenes were identified (mean 78 seconds and s.d 22.3 seconds), 14 scenes were less than 80 seconds, and 18 scenes were greater than 80 seconds.

The dataset was not annotated manually to serve as ground truth, since previous work has emphasized that human event segmentation is inherently subjective and shows systematic variability across observers (Zacks, 2020). Individual differences in where people place boundaries are linked to factors such as prior knowledge or observer’s interpretation (Radvansky & Zacks, 2017). Therefore, we focused on comparing the proposed ensemble segmentation against segmentations from each single LLM. These scenes’ timings were also validated by a human and purpose of this validation was only to verify that the segmented scenes by LLMs actually matched with the stimuli, rather than to define a new set of “ground-truth” boundaries/scenes.

The selected LLMs differ in training data composition, details can be found in Supplementary Table 1. This variety in training approaches supports the ensemble use of multiple LLMs to reduce single-model bias and increase robustness in tasks such as narrative event segmentation. The scenes segmented by the Algorithm 1 was then used to calculate the FC matrices that were particularly useful in analyzing how memory recall is modulated by specific narrative events. We tried to asscociate the recall scores with the stimuli. The use of LLM-driven segmentation in computing FC matrices provides a more nuanced understanding of how cognitive processes unfold during narrative comprehension. This, in turn, will have an impact on classification performance, as the accurate identification of scene windows corresponding to memory recall scores will lead to better separation between classes.

### Classification

Four different datasets were used in this study to evalute the performace of the classification across different classifiers for memory recall. The classification performance was evaluated across three components derived from the decomposition of the FC matrices: the original FC matrices, low-rank matrices, and sparse matrices. The purpose of this analysis is to assess whether the decomposition could reveal distinct patterns in the data that improves the classification performance. Both high and low-recall scenes are present in every story indicating that the labels do not trivially collapse “easy” stories into one class and “difficult” stories into another. Story 1 has 360 samples, story 2 has 450 samples, story 3 has 360 samples, and story 4 has 270 samples, with each story having low-recall labels proportions of 28%, 28%, 22% and 21%, respectively. The overall dataset comprises 1440 samples, with a ratio of high recall to low recall distribution of 1:3. A Leave-One-Out Cross-Validation (LOOCV) (Gupta & Vig, 2019) strategy was incorporated to ensure the robustness of the results.

Two well-known networks, auditory and DMN were employed for classification purposes, leveraging the rich information contained in their FC matrices, decomposed using low-rank algorithms. Three classifiers - SVM, logistic regression, and random forest (RF) - were utilized to explore the potential of these networks in predicting memory recall scores. Notably, the actual FC matrix exhibited relatively similar classification accuracy compared to its sparse and low-rank component matrices.

The FC matrix demonstrated moderate performance in both the DMN and auditory networks. In the DMN network Fig. 2, the FC matrix achieved an accuracy of nearly 70% when compared to randomly assigned labels. Similarly, in the auditory network Fig. 3, the FC matrix also reached approximately 70% accuracy under the similar conditions. These baseline performances provided a basis for comparing the results for further analyses involving low-rank and sparse matrices. Importantly, label shuffling experiments revealed a significant drop in accuracy to around 52%, highlighting the alignment of memory recall labels with fMRI data derived from scene onset and offset timings computed by LLMs.

We have also done classification using individual LLM based scene segmentation. The mean and standard deviation (std) of classification accuracy of the individual LLMs based scene segmentation is shown in Fig. 4 as well as in Supplementary Table 3. The results showed that the proposed scene-segmentation framework yielded better and consistent accuracy across all three classifiers. In contrast, individual LLMs exhibited variable performance, with only one model (claude) achieving a marginal improvement of 1–2% in accuracy. This highlights the robustness and generalizability of using the proposed scene-segmentation framework over the individual models. When incorporating low-rank and sparse components into the analysis, the DMN network showed promising results.

The low-rank DMN component achieved a slightly higher classification accuracy (76.7%) when combined with RF classifiers, with 95% confidence intervals (CIs) of [71.3, 82.1]. This exceeds the accuracy obtained using full FC of 72% with 95% CIs of [67.3, 76.7]), reflecting an absolute improvement of 4.7 percentage points. A paired comparison across repeated runs confirmed that this gain is statistically reliable ($\bigtriangleup \approx 0.047, t=19.4, p<10^{-4}$). Beyond accuracy, the low-rank DMN features yielded a balanced accuracy of 0.58 and an AUC of 0.573.

For the Auditory network, RF classifiers also performed slightly better with low-rank features compared to other model variants, though FC and sparse representations showed similar performance overall. The low-rank Auditory network achieved 71% accuracy with 95% CIs of [66, 75], with a balanced accuracy of 0.55 and an AUC of 0.53. By comparison, RF with FC achieved 70% accuracy with 95% CIs of [65, 74], a balanced accuracy of 0.56, and an AUC of 0.53.

**Fig. 5:**
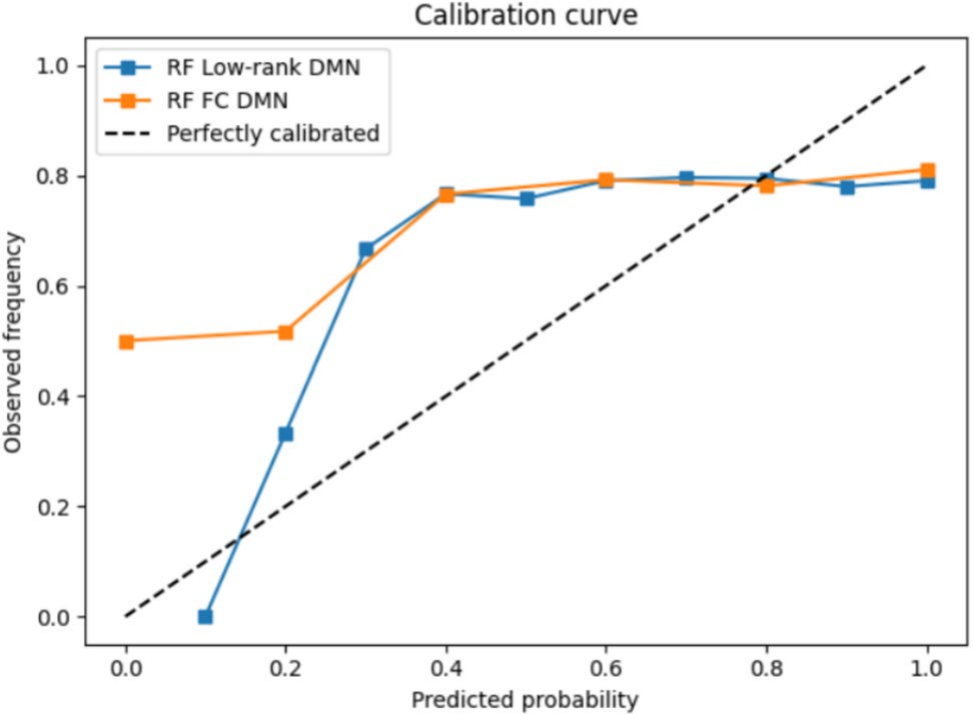
Calibration curve for DMN network

**Fig. 6:**
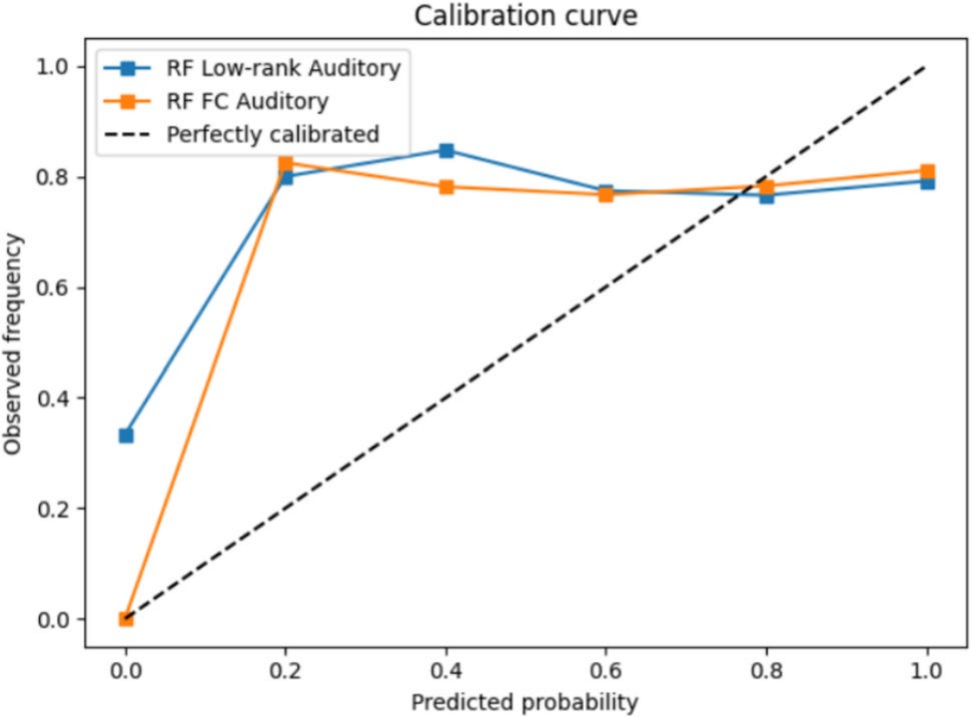
Calibration curve for Auditory network

The calibration curves for DMN and auditory network are given in Figs. 5 and 6 respectively. The calibration curves for the auditory network have higher deviation at lower predicted probabilities compared to DMN network. The curves are not well calibrated since the data set is imbalanced. Overall, these findings suggest that low-rank DMN connectivity provides a small but reliable improvement over raw FC for recall classification.

### Discussion

Brain network dynamics during naturalistic stimuli can differ significantly between high-order networks such as DMN and primary auditory/sensory networks. DMN is associated with internal-oriented cognition and integrates information over long timescales (Simony et al., 2016a). In DMN the connectivity patterns fluctuate substantially and reflect a mix of ongoing intrinsic processes and subtle effects. Classic findings indicate DMN activity and connectivity are only weakly modulated by external stimuli, however, the coherent narratives do induce reliable DMN connectivity patterns, but these patterns are complex – specific to each story segment’s meaning and requiring cross-subject analysis to observe clearly (Simony et al., 2016a). For example, when a story is scrambled (loses narrative coherence), DMN inter-regional correlations become far less reliable suggesting the DMN does engage with high-level narrative structure, but in a nuanced way, as seen in Fig. 2 against logistic and SVM classifiers. The DMN’s stimulus-driven changes are real but multi-faceted and interwoven with intrinsic activity, leading to a lower signal-to-noise ratio and more complex patterns in DMN features (Simony et al., 2016a).

By contrast, the Auditory network (and other sensory networks) responds more directly and uniformly to external auditory stimuli. During an auditory story, regions in the auditory network will show synchronized activation and connectivity with time-locked to the sound and speech content (Ting et al., 2021). When different individuals process the same narrative or movie, sensory networks exhibit highly synchronized connectivity dynamics across subjects, much more than during rest or incoherent stimuli (Türker et al., 2023). This indicates a strong, consistent stimulus-induced signal in auditory network -effectively captured essential features, enhancing classification performance as seen by auditory network results Fig. 3 for low-rank component.

The low-rank and sparse analyses revealed limitations in capturing patterns related to memory recall studies, especially when utilizing logistic and SVM classifiers. However, it still offers a valuable complement to traditional FC matrix analysis, warranting further exploration into its potential benefits for improving classification models. The random shuffling of labels led to a significant drop in accuracy across all three cases, affirming the alignment of recall labels with the corresponding fMRI data based on scene onset and offset timings computed by LLM-based algorithm. Taken together, the above-chance balanced accuracy and AUC, the robustness of the low-rank results, and the sensitivity of performance to label shuffling indicate that the proposed pipeline captures the relationships between narrative structure, brain connectivity, and memory. This also validates the hypothesis that associations between memory recall and stimulus presentation are reflected in brain responses. In practical terms, even marginal improvements in accuracy could be valuable, suggesting applications where every little bit of improvement matters. However, these results also highlight areas for deeper exploration—understanding why certain components perform better and optimizing their use in classification tasks.

## Conclusion and future work

In this study, we showed that use of LLMs for text-based scene segmentation can accurately segment naturalistic narrative stimuli. This approach eliminates subjective bias of human annotators and reduces LLM-specific biases by combining the output of multiple LLMs. The ensemble based LLM segmentation of scenes’s timings was used to construct FC features from DMN and auditory networks for scene level recall classification. We also applied low-rank decomposition on raw FC and found that the low rank DMN network, when combined with a RF classifier yielded a modest but statistically reliable improvement in accuracy over raw FC.

At the same time, few limitations undermine these findings. Overall discriminability between high and low recall scenes remained moderate, and a class imbalance likely constrained performance despite our use of appropriate metrics. The analysis focused on four stories of narrative fMRI datasets, two well-defined networks, and relatively simple classifiers, and the generalizability of the approach to other modalities (i.e video stimulus) and networks is needed.

Future work could extend this framework to additional brain networks and modalities, explore richer multi-model behavioral labels, and investigate more advanced modeling approaches —like foundation models to better capture how shared and idiosyncratic neural patterns support memory for naturalistic events. In addition, the low-rank decomposition algorithm could be further optimized, for example by incorporating non-linear methods, to capture a broader range of connectivity patterns related to recall.

## Supplementary Information

Below is the link to the electronic supplementary material.

Supplementary file 1 (pdf 105 KB)

## Data Availability

No datasets were generated or analysed during the current study.

## Footnotes

1 https://platform.openai.com/tokenizer
