# Supplementary material for: Classification of decomposed neural data in memory networks and LLM-based stimuli processing
Source: Brain Imaging Behav. 2026 Apr 18;20(3):75. doi: 10.1007/s11682-026-01148-4 (PMC13091883; doi:10.1007/s11682-026-01148-4)
Supplement: Supplementary file 1 — (pdf 105 KB) [file 11682_2026_1148_MOESM1_ESM.pdf]

Table 1: Supplementary Table. Default Mode Network (DMN) and Auditory Network ROIs MNI coordinates derived from (Seitzman et al., 2020). Pink color cells belong to the Auditory Network and green color belong to DMN.

**Sr. no**	**x**	**y**	**z**	Radius (mm)	Brain Cortices*
1	-52.92	-21.83	22.97	5	1
2	-60.48	-25.22	13.82	5	1
3	-55.22	-9.42	11.73	5	1
4	-49.77	-34.36	25.74	5	1
5	-49.14	-26.3	5.18	5	1
6	-38.43	-33.34	16.98	5	1
7	43.45	-22.93	19.85	5	2
8	59.4	-17.34	28.69	5	2
9	31.75	-26.33	12.91	5	2
10	55.96	-5.03	13.25	5	2
11	57.88	-15.62	7.49	5	2
12	65.43	-33.2	19.97	5	2
13	-68.47	-22.66	-15.74	5	1
14	-68.3	-41.41	-5.14	5	1
15	-57.75	-29.7	-3.94	5	1
16	-55.72	-12.96	-10.24	5	1
17	-52.89	2.55	-27.06	5	1
18	-49.3	-42.15	0.83	5	1
19	-45.79	-60.69	20.85	5	1
20	-46.17	31.26	-13.03	5	1
21	-44.45	-64.64	34.78	5	1
22	-40.5	-75.27	25.8	5	1
23	-39.05	-74.95	43.72	5	1
24	-35.36	19.86	50.8	5	1
25	-19.78	45.07	39.48	5	1
26	-20.16	63.65	19.39	5	1
27	-17.65	63.19	-9.17	5	1
28	-16.4	28.52	53.05	5	1
29	-12.6	-39.64	0.93	5	1
30	-11.29	-56.2	15.6	5	1

Continued on next page

Sr. no	x	y	z	Radius (mm)	Brain Cortices*
31	-10.09	39.09	52.29	5	1
32	-10.33	54.63	38.71	5	1
33	-11.06	44.62	7.61	5	1
34	-7.55	48.08	23.18	5	1
35	-6.84	-54.9	27.05	5	1
36	-7.04	50.82	-1.29	5	1
37	-2.94	-48.79	12.87	5	1
38	-2.2	-36.68	43.85	5	1
39	-3.06	44.41	-9.46	5	1
40	-2.06	37.85	36.34	5	1
41	-2.5	41.7	16.05	5	1
42	-33.93	-38.06	-15.6	5	1
43	5.55	66.69	-3.55	5	2
44	5.91	-58.82	35.45	5	2
45	5.94	54.42	16.18	5	2
46	6.11	63.98	21.96	5	2
47	7.94	-48.37	30.57	5	2
48	7.51	42.49	-5.35	5	2
49	8.36	47.59	-15.18	5	2
50	8.8	54.23	3.45	5	2
51	10.77	-53.83	17.09	5	2
52	12.73	54.87	38.19	5	2
53	13.08	29.99	58.65	5	2
54	12.25	35.63	20.3	5	2
55	15.12	-63.09	25.98	5	2
56	22.11	39.21	38.9	5	2
57	23.33	33.07	47.68	5	2
58	43.43	-72.21	28	5	2
59	45.64	16.2	-30.02	5	2
60	46.68	-50.08	28.76	5	2
61	49.26	35.47	-12.2	5	2
62	52.04	-59.37	35.52	5	2
63	52.16	-2.43	-16.4	5	2
64	51.9	6.81	-29.61	5	2

Continued on next page

**Sr. no**	**x**	**y**	**z**	Radius (mm)	Brain Cortices*
65	64.64	-11.8	-19.3	5	2
66	64.8	-30.55	-8.7	5	2
67	-25.24	-38.78	-2.01	4	3
68	25.08	-37.18	-2.16	4	3
69	3.42	-7.79	8.23	4	6
70	-2.88	-9.96	8.5	4	6

* Brain Cortices: 0=cortexMid, 1=cortexL, 2=cortexR, 3=hippocampus, 4=amygdala, 5=basalGanglia, 6=thalamus, 7=cerebellum.

Table 2: Overview of Training Data and Domain Characteristics of Five State-of-the-Art LLMs

**Model**	**Training Data Sources**	**Key Domains / Corpora**	**Multilingual Coverage**	**Special Focus Areas**	**Knowledge Cutoff**
**Claude 3 Opus (A. Anthropic, 2024) (Anthropic, 2024)**	1. Proprietary mix of public web data 2. Third-party licensed data, 3. Data labeling 4. Internally generated synthetic data	General web text, curated instructional content	Some multilingual data, not emphasized	Safety, helpfulness, and ethical alignment; no code emphasis	August-2023
**Perplexity (GPT-4 Turbo) (O. AI, 2024)**	1. Large-scale mix of public internet data 2. Licensed datasets 3. Books and images.	Web text, books, licensed data, Reddit, code, images	Limited multilingual support	Multimodal (vision + text), fine-tuned with human feedback	April-2023
**ChatGPT-4 (O. AI, 2024)**	1. Similar to GPT-4 Turbo 2. Multimodal public + licensed data	Web text, licensed datasets, books, code, images	Limited multilingual coverage	Multimodal input, fine-tuned with RLHF	April-2023
**LLaMA 3.1 (M. AI, 2024)**	1. Over 15 trillion tokens from public sources only. 2. No proprietary or licensed data.	Wikipedia, books, GitHub (code), scientific content	~5% of data in 30+ languages	Strong on code, reasoning, and multilingual filtering	Mid-2023
**MS-Copilot (Phi-3 14B) (Abdin et al., 2024)**	1. 3.3T tokens from filtered public web data. 2. Synthetic educational content	Textbooks, step-by-step QA, scientific/math instruction	Multilingual not emphasized	Small, efficient model for math, logic, structured reasoning	2023

Table 3: Mean and Standard deviation (Std) of classification accuracies for individual LLMs based scene parcellation.

Auditory									
	**Individual LLMs (Std)**			**Individual LLMs (Mean)**			**Proposed Framework**		
	Logict	SVM	RF	Logict	SVM	RF	Logist	SVM	RF
**Accuracy**	2.1	2.1	2.7	63.4	61	68.2	69	64	70
**F1.score**	2.2	2.1	2.4	63.6	62	64.4	69	65	67

DMN									
	**Individual LLMs (Std)**			**Individual LLMs (Mean)**			**Proposed Framework**		
	Logict	SVM	RF	Logict	SVM	RF	Logist	SVM	RF
**Accuracy**	2.3	1.9	2.2	67.2	65.4	70	72	72	72
**F1.score**	2.0	2.4	2.4	65.4	65.2	66	70	70	68

References

Abdin, M., Aneja, J., Awadalla, H., Awadallah, A., Awan, A. A., Bach, N., Bahree, A., Bakhtiari, A., Bao, J., Behl, H., et al. (2024). Phi-3 technical report: A highly capable language model locally on your phone. *arXiv preprint arXiv:2404.14219*.

AI, M. (2024). Meta-llama-3 [[Accessed 22-10-2024]].

AI, O. (2024). How chatgpt and our foundation models are developed [[Accessed 22-10-2024]].

Anthropic. (2024). Anthropic's Transparency Hub — anthropic.com [[Accessed 22-10-2024]].

Anthropic, A. (2024). The claude 3 model family: Opus, sonnet, haiku. *Claude-3 Model Card*, *1*, 1.

Seitzman, B. A., Gratton, C., Marek, S., Raut, R. V., Dosenbach, N. U., Schlaggar, B. L., Petersen, S. E., & Greene, D. J. (2020). A set of functionally-defined brain regions with improved representation of the subcortex and cerebellum. *Neuroimage*, *206*, 116290.
